# Life-Threatening Hypotension in a Brain-Injured, Multi-Trauma Patient With Unilateral Adrenal Gland Damage: How a Single Hydrocortisone Dose Revealed Relative Corticosteroid Insufficiency

**DOI:** 10.7759/cureus.32843

**Published:** 2022-12-22

**Authors:** Georgios E Zakynthinos, Paris Zygoulis, Alexandra Tsikrika, Vasiliki Tsolaki

**Affiliations:** 1 Cardiology, 3rd Department of Cardiology, Athens Chest Disease Hospital "Sotiria", Athens, GRC; 2 Critical Care, University Hospital of Larissa, University of Thessaly, Faculty of Medicine, Larissa, GRC; 3 Radiology, University Hospital of Larissa, University of Thessaly, Faculty of Medicine, Larissa, GRC

**Keywords:** intensive care, corticosteroids, adrenal gland injury, resistant shock, adrenal insufficiency (ai)

## Abstract

We report a case of a multi-trauma, brain-injured young patient with unilateral adrenal gland injury presenting with refractory shock. Acute adrenal insufficiency was revealed after an abrupt hemodynamic response to a corticosteroid; the resistant shock was quickly resolved with IV hydrocortisone. Although available data do not support the use of empiric steroids in trauma patients (with or without brain injury), this case demonstrates that adrenal insufficiency must be considered in the differential diagnosis when shock exists; adrenal gland injury, even unilateral, may play an additional factor. In these cases, an urgent decision is required in order to influence the outcome.

## Introduction

The incidence of adrenal injury after trauma is very rare [1-3]. Identification of adrenal injury can be of critical importance in patients with bilateral adrenal injury, which may lead to acute adrenal insufficiency and death, whereas unilateral adrenal trauma is often asymptomatic and masked by injuries to other organs [1,3,4]. However, when unilateral adrenal trauma is associated with multiple injuries including brain trauma, as in our case, critical illness‑related corticosteroid insufficiency (CIRCI) may be present; despite the importance of timely action, criteria for the diagnosis are not well established. This article was previously posted to the Research Square preprint server on June 23, 2021. 

## Case presentation

**Figure 1:**
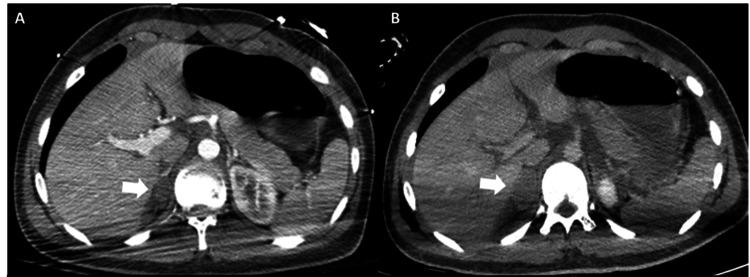
Abdominal CT findings A. Abdominal CT scan on admission: the arrow indicates a hypodense lesion located in the right adrenal gland, suggesting hematoma (2.48 x 1.61 cm).b. B. The CT scan shows that the right adrenal gland hematoma dimension increased nine hours after admission (4 x 2.7 cm). Diffuse perihepatic and peripancreatic hemorrhagic fluid collection can also be seen.

A 16-year-old male presented to the emergency department after a motor vehicle accident with multi-trauma; he was comatose (Glasgow Coma Scale (GCS) of 5), severely hypotensive, and was immediately intubated. Brain CT revealed extensive contusions, subdural hematoma, and brain edema, while chest and abdominal computed tomography (CT) scan revealed multiple lung contusions, pneumomediastinum, and pneumothorax with subcutaneous emphysema needing a chest tube insertion, multiple fractures (involving the C2 (Type I), C5, C7, left clavicle, multiple right ribs), and hemorrhagic collection in Morrison’s pouch, extended to the right perinephric space. There was a hypodense lesion in the adrenal gland suggesting a hematoma (2.48 x 1.61 cm), with periadrenal fat stranding (Figure 1A). Multiple bleeding skin lacerations were also present.

Laboratory findings included hematocrit (Hct) of 28% and white blood cells (WBC) of 10,500 cells/μL platelets (PLT) 143,000/μL; coagulation and liver function tests were normal. Two units of red blood cells (RBC), two units of fresh frozen plasma (FFP), and two platelet units were administered. The patient was transferred to the operating room, where an intraparenchymal catheter for intracranial pressure (ICP) monitoring was inserted and a craniectomy was performed.

Postoperatively, the patient was admitted, just after midnight, to the ICU under sedation. He presented severe circulatory shock (noradrenaline dose of 1,86 μg/kg/min); three RBC units and fluids (4 liters in 9 hours) for resuscitation were administered; however, shock was not reversed (noradrenaline increased to 2 μg/kg/min, lactate 1.8-2.1 mmol/L, although Hct was stabilized to 34 g/dl). Echocardiography was also performed, excluding signs of acute heart failure, stress cardiomyopathy, signs of obstructive shock, or cardiac tamponade. APACHE and SOFA scores upon admission were 29 and 10 respectively.

Empiric hydrocortisone (100 mg intravenously) was administered for suspected adrenal insufficiency after a blood sample for cortisol levels was drawn (the adrenocorticotropic hormone and corticotropin (ACTH) stimulation test was not performed). An abrupt improvement in hemodynamics was noted: noradrenaline dose was reduced by half (1 μg/ kg/min) in less than one hour, and almost became insignificant during the next eight hours, with lactate levels at 0.9 mmol/L.

Hydrocortisone administration was continued at a dose of 100 mg three times daily, for three days, 150 mg for two days, and was tapered over the next four days. The fluid balance was restored after the first day. Baseline cortisol levels (results received after a three-day delay) were 11.45 μg/dl. Intracranial pressure (ICP) was steadily less than 20 mmHg.

Adrenal hematoma dimensions had increased (4 x 2.7 cm), as seen in the abdominal CT scan performed nine hours after admission (Figure 1B). Twenty days later, a follow-up CT scan revealed regression of the hematoma. His remaining ICU course was complicated by fever and sepsis and remained in the ICU for 41 days. He was discharged to a rehabilitation center and his brain function was gradually restored (GCS 15), although extensive spasticity remained.

Informed written consent was obtained from the patient's next of kin.

## Discussion

In our multi-trauma, brain-injured patient with a unilateral adrenal gland injury, presenting with refractory shock, a bolus hydrocortisone dose uncovered critical illness-related corticosteroid insufficiency (CIRCI). Exogenous hydrocortisone administration balanced the cortisol levels needed to manage the patient's stress disorder, thus leading to an abrupt weaning from vasopressors (initially high noradrenaline dose was almost stopped after hydrocortisone administration) and reduced the need for fluid administration.

CIRCI may be common in severe trauma patients and is associated with uncontrolled inflammation, vasopressor dependency, and poor clinical outcomes [2,5]. Independent of adrenal gland trauma (AGT) presence, the coexistence of AGT may deteriorate the clinical status.

Therefore, the present case raises the following questions:

Firstly, is there a real cut-off level of plasma cortisol to define CIRCI in severe cases and especially in trauma? Is trauma severity a key factor? Secondly, does unilateral AGT participate in CIRCI? Thirdly are there any clinical indications in trauma patients with shock to administer a bolus corticosteroids dose in order to diagnose CIRCI?

CIRCI occurs across a broad spectrum of critical illnesses. Most guidelines are extrapolated from studies in sepsis [6]. However, even in septic shock, the most studied pathology, there is no standard method to diagnose CIRCI. The task force of the Society of Critical Care Medicine (SCCM) and the European Society of Intensive Care Medicine (ESICM) were unable to reach an agreement on a single test that can reliably diagnose CIRCI, although they suggest that clinicians may use a random plasma cortisol of <10 μg/dl for diagnosis [6,7].

Therefore, cortisol levels, if CIRCI guidelines could be extrapolated to trauma, of 11.45 μg/dL in our patient could initially be considered sufficient. However, stress degrees may be completely different depending on injury severity and the etiology of critical illness. Gannon et al. defined adrenal insufficiency in critically ill trauma patients as serum cortisol less than 25 mcg/dL, [7] thus diagnosing occult adrenal insufficiency in over 50% of their patients. Recently multi-trauma patients were categorized into three groups, namely severely low, relatively low, and normal cortisol levels, when serum cortisol levels were ≤15 µg/dL, 15.01-25 µg/dL, or >25 µg/dL respectively, revealing the confusion that prevails concerning cortisol levels when defining relative adrenal insufficiency [8].

Adrenal gland trauma (AGT) is often a result of blunt trauma, reported most commonly in association with associated injuries to the ribs, thorax, spine, kidney, spleen, and liver [1-3]. Therefore, AGT is associated with high injury severity and mortality rates up to five times higher than non-AGT trauma [3]. Usually, AGT is a coincidental finding on diagnostic imaging; therefore, the overall incidence in trauma patients has not been well characterized, although a rate ranging from 0.03 to 4,95% has been reported [1-3]. Life-threatening adrenal insufficiency may follow [5].

The SCCM and ESICM suggest against the use of corticosteroids in major trauma, despite the low quality of evidence [6]. Yet no recommendations exist for major trauma associated with adrenal gland injury (bilateral or unilateral) and shock [6]. Moreover, low Hct and hemorrhage followed by shock may cover adrenal insufficiency.

Cortisol levels were clearly insufficient at this grade of severity in our multi-trauma with brain injury patient. Probably, isolated AGT contributed to circulatory collapse, suggesting that serum cortisol levels, indicating adrenal insufficiency, may vary depending on the degree of stress induced by the injury.

We did not perform a cosyntropin test. To date, this diagnostic criterion has not been adopted in routine practice [6]. Even in sepsis patients, the latest surviving sepsis campaign guidelines suggest against using the ACTH stimulation test in the case of select patients that may be treated with hydrocortisone [9]. Yet, delta cortisol (change in baseline cortisol of <9 μg/dl after cosyntropin administration) may have a clinical application when cortisol plasma levels are very low, as cortisol levels below 18 μg/dl after ACTH stimulation test may indicate adrenal insufficiency [10]. However, very low plasma cortisol levels are not observed in severe trauma patients [7,8].

To date, there is insufficient data to support the use of hemodynamic response to a single hydrocortisone dose (50-300 mg) as a reliable test for the diagnosis of CIRCI [6]. However, in our patient there was an abrupt hemodynamic improvement, enabling the weaning from vasopressors in a few hours and normalizing lactate levels, clearly indicating CIRCI; the patient’s Hct had been stabilized, and he was not responding to fluid resuscitation.

Although hydrocortisone has been found to improve the vasopressor response to norepinephrine at least in septic patients, this effect is more marked in patients with CIRCI [6]. Therefore, we believe that a single dose of corticosteroids (followed by IV hydrocortisone for a few days after a positive result) helped to reach a diagnosis of CIRCI in our multi-trauma AGT patient. Moreover, this test seems to be safe without disturbing immune response; even hydrocortisone therapy did not affect or even decreased infections (hospital-acquired pneumonia) in multiple trauma patients with or without head trauma, respectively [11,12]. 

## Conclusions

Life-threatening CIRCI must be considered in a multi-trauma hypotensive patient with AGT. As there are no reliable tests to diagnose CIRCI, a dose of hydrocortisone may reveal relative corticosteroid insufficiency in a hemodynamically unstable patient not responding to resuscitation.
